# Supplementary material for: Expression Profile of microRNAs during Development of the Hypopharyngeal Gland in Honey Bee, Apis mellifera
Source: Int J Mol Sci. 2022 Oct 26;23(21):12970. doi: 10.3390/ijms232112970 (PMC9658247; doi:10.3390/ijms232112970)
Supplement: Supplementary file 1 [file ijms-23-12970-s001.zip › Table S1-Person correlations of the nine samples.pdf]

Table S1. Person correlations of the nine samples.

	NEW1	NEW2	NEW3	Nurse1	Nurse2	Nurse3	Forager1	Forager2	Forager3
NEW1	1								
NEW2	0.9705	1							
NEW3	0.9916	0.9769	1						
Nurse1	0.6847	0.5303	0.6475	1					
Nurse2	0.6884	0.5416	0.6446	0.9884	1				
Nurse3	0.6599	0.5017	0.619	0.9941	0.9866	1			
Forager1	0.7152	0.5702	0.6743	0.9835	0.9915	0.9784	1		
Forager2	0.7566	0.6234	0.7217	0.9683	0.9819	0.9636	0.9889	1	
Forager3	0.9248	0.8321	0.9084	0.8669	0.8682	0.855	0.8904	0.9234	1
