# Supplementary material for: Expression Profile of microRNAs during Development of the Hypopharyngeal Gland in Honey Bee, Apis mellifera
Source: Int J Mol Sci. 2022 Oct 26;23(21):12970. doi: 10.3390/ijms232112970 (PMC9658247; doi:10.3390/ijms232112970)
Supplement: Supplementary file 1 [file ijms-23-12970-s001.zip › Table S7-Primers for stem-loop RT-PCR and RT-qPCR.pdf]

Table S7. Primers for stem-loop RT-PCR and RT-qPCR

	miRNA	Primer Sequence (5' to 3')
stem-loop RT-PCR	novel-miR-109	F: AACACGTGAACCCGTAGATCCG<br>RT Primer: GTCGTATCCAGTGCAGGGTCCGAGGTATTCGCACTGGATACGACCACAAG
	novel-miR-45	F: GGCGCGTAATACTGTCAGGTAAAG<br>RT Primer: GTCGTATCCAGTGCAGGGTCCGAGGTATTCGCACTGGATACGACACATCT
	novel-miR-135	F: AGCCAGCGTCAGTCTTTTTCTCT<br>RT Primer: GTCGTATCCAGTGCAGGGTCCGAGGTATTCGCACTGGATACGACATAGGA
	novel-miR-34	F: AACACGCTATCACAGCCAGCTT<br>RT Primer: GTCGTATCCAGTGCAGGGTCCGAGGTATTCGCACTGGATACGACGCTCAT
	novel-miR-53	F: CCGCCTAGGAACTTCATACCG<br>RT Primer: GTCGTATCCAGTGCAGGGTCCGAGGTATTCGCACTGGATACGACAGAGCA
	novel-miR-137	F: CGACAGGCAAGAGAGCTATCCAT<br>RT Primer: GTCGTATCCAGTGCAGGGTCCGAGGTATTCGCACTGGATACGACACTGTC
	novel-miR-11	F: AACCGGTTTGTTCGTTCGGCT<br>RT Primer: GTCGTATCCAGTGCAGGGTCCGAGGTATTCGCACTGGATACGACTAACTC
	Universal-R	ATCCAGTGCAGGGTCCGAGG
RT-qPCR	ame-miR-34-5p	F: GCAGTGGCAGTGTTGTTAG<br>R: CCAGTTTTTTTTTTTTTTTCAACCAG
	novel-miR-107	F: GCAGTTCTCTTTGGTTGTTAC<br>R: GGTCCAGTTTTTTTTTTTTTTTAGTG

RT-qPCR	ame-miR-317-3p	F: GAACACAGCTGGTGGTATC<br>R: GTCCAGTTTTTTTTTTTTTTTACTGAG
	ame-miR-263a-5p	F: AGGTAAATGGCACTGGAAGA<br>R: GGTCCAGTTTTTTTTTTTTTTTGTG
	ame-miR-3785-3p	F: ACCCTGTAACGTCCTGAG<br>R: GGTCCAGTTTTTTTTTTTTTTTAGTC
	novel-miR-124	F: AGGAAGCTCGTCTCTACAG<br>R: GGTCCAGTTTTTTTTTTTTTTTAGATAC
	ame-miR-2796-3p	F: GCCGGCGGAAACTAC<br>R: TCCAGTTTTTTTTTTTTTTTGCAAG
	ame-miR-29b-3p	F: CGCAGTAGCACCATTTGA<br>R: GGTCCAGTTTTTTTTTTTTTTTACTGA
	ame-miR-315-5p	F: GCAGTTTTGATTGTTGCTCAGA<br>R: GGTCCAGTTTTTTTTTTTTTTTGCT
	novel-miR-120	F: CAGTATCACAGCCATTTTTGAC<br>R: GGTCCAGTTTTTTTTTTTTTTTAATCG
	novel-miR-23	F: GCAGTCGGTAAGCAGAGT<br>R: GTCCAGTTTTTTTTTTTTTTTGGTCT
	novel-miR-80	F: TGCGTCGTCTGATCGT<br>R: GTCCAGTTTTTTTTTTTTTTTAGAAACG
	ame-miR-210-3p	F: AGTTGTGCGTGTGACAG<br>R: GGTCCAGTTTTTTTTTTTTTTTAGC

RT-qPCR	novel-miR-99	F: GCAGTCTTTGGTTATCTAGCTG<br>R: GGTCCAGTTTTTTTTTTTTTTTCATAC
	novel-miR-95	F: GGCAAGATGTCGGCATAG<br>R: GGTCCAGTTTTTTTTTTTTTTTCAG
	U6	F: GGAACGATACAGAGAAGATTAGC<br>R: TGGAACGCTTCACGAATTTGCG
