# Supplementary material for: Expression Profile of microRNAs during Development of the Hypopharyngeal Gland in Honey Bee, Apis mellifera
Source: Int J Mol Sci. 2022 Oct 26;23(21):12970. doi: 10.3390/ijms232112970 (PMC9658247; doi:10.3390/ijms232112970)
Supplement: Supplementary file 1 [file ijms-23-12970-s001.zip › Table S8-3íSUTR fragments used in the dual-luciferase-reporter assay.pdf]

Table S8. 3'UTR fragments used in dual-luciferase-reporter assay

name	sequence
LOC410685-WT	TTATGTCGCGTATATATCCGTATTCTCGAATATATATCGAACTCGTCGGTGTTTCGAGTGGGC<br>TAGAATCGTGGGGTCATCATCTTGTGTGTCTATCGCACGAGATGATAAGAAAAGGGGGAG<br>GGAAATAGGGAGGAACTCGATGTCGACTCGAACAAGATGAAAAAGAATGAAACAGCCTT<br>GTACATACCGTGTAAGCTTAAGTTTGCTCGTTAAGTAACACAATTGCGAGATTCGAGTTTTT<br>CATTTTTCATATATATAAATATAAATATATATATAT
LOC410685-MUT	TTATGTCGCGTATATATCCGTATTCTCGAATATATATCGAACTCGTCGGTGTTTCGAGTGGGC<br>TAGAATCGTGGGGTCATCATCTTGTGTGTCTATCGCACGAGATGATAAGAAAAGGGGGAG<br>GGAAATAGGGAGGTTGAGCTTCAGCTCTGCTTGTTCATGAAAAAGAATGAAACAGCCTTG<br>TACATACCGTGTAAGCTTAAGTTTGCTCGTTAAGTAACACAATTGCGAGATTCGAGTTTTTC<br>ATTTTTCATATATATAAATATAAATATATATATAT
LOC725318-WT	ATCGAGTGGAATGTATGATTTAGAAAGACTCGAGTAGCGATCGTTTCCAAGCCTCGTCTCT<br>CGATCGATTAACTTTCTATGATCGAAGCTCGTCGTAAAAAAGAAAGAAAAATAGAAAGAA<br>AAGGAACGAGAGAGATGACTCGAGCTTTTTGAGCGAACGAAGAGTTGAGTATCGTGGTG<br>CATTTGAACGTACGTATGGTTTAATGATTCCGATTCCAGTTTTGAGAAATGAGAATACACAA<br>CGTGAATTATTTTGAAACTTTCGATTCTTCAAATTCTA

LOC725318-MUT	ATCGAGTGGAATGTATGATTTAGAAAGACTCGAGTAGCGATCGTTTCCAAGCCTCGTCTCT<br>CGATCGATTAACTTTCTATGATCGAAGCTCGTCGTAAAAAAGAAAGAAAAATAGAAAGAA<br>AAGGAACGAGAGAGAACTGAGCTCGATTTTCTCGCTTGCTTGAGTTGAGTATCGTGGTGC<br>ATTTGAACGTACGTATGGTTTAATGATTCCGATTCCAGTTTTGAGAAATGAGAATACACAAC<br>GTGAATTATTTTGAAACTTTCGATTCTTCAAATTCTA
