# Supplementary figures and images for: Expression Profile of microRNAs during Development of the Hypopharyngeal Gland in Honey Bee, Apis mellifera
Source: Int J Mol Sci. 2022 Oct 26;23(21):12970. doi: 10.3390/ijms232112970 (PMC9658247; doi:10.3390/ijms232112970)

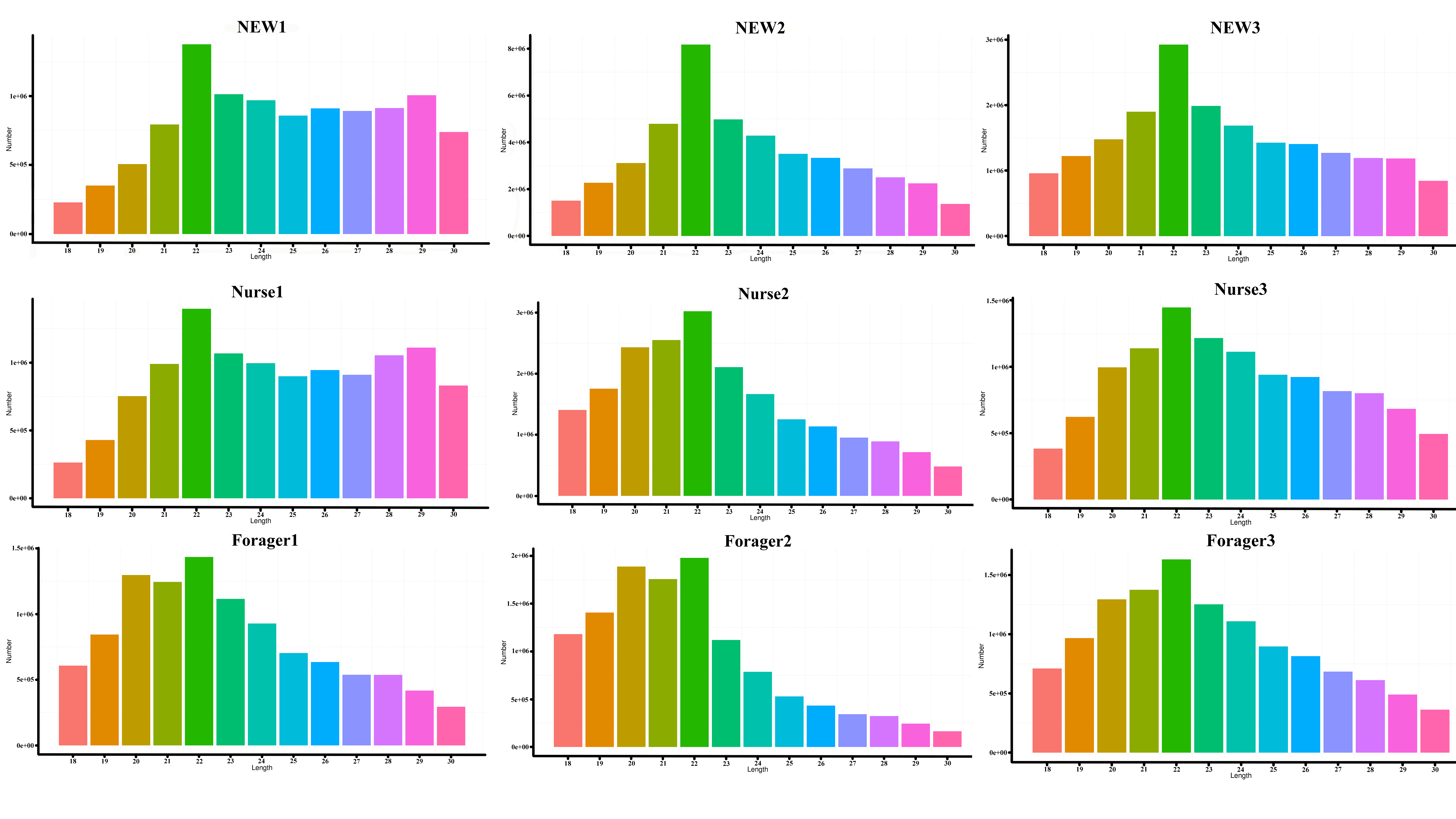

Supplement: Supplementary file 1 [file ijms-23-12970-s001.zip › Figure S1-Length distribution of reads in the nine samples.png]
